# The current status of trauma care for older adults in Saudi Arabia

**DOI:** 10.3389/fmed.2024.1505913

**Published:** 2024-12-13

**Authors:** Naif Harthi, Steve Goodacre, Fiona C. Sampson

**Affiliations:** ^1^Emergency Medical Services Programme, Department of Nursing, College of Nursing and Health Sciences, Jazan University, Jazan, Saudi Arabia; ^2^Sheffield Centre for Health and Related Research, University of Sheffield, Sheffield, United Kingdom

**Keywords:** trauma, trauma care, older adults, prehospital care, Saudi Arabia

## Abstract

The aging population in Saudi Arabia presents unique challenges to the trauma care system, especially in prehospital settings. This narrative review aims to assess the current status of trauma care for older adults in Saudi Arabia, focusing on the implications of aging, gaps in the trauma system, and the role of emergency medical services. The global rise in life expectancy has increased the incidence of injuries among older adults, leading to a greater burden on healthcare systems. The review highlights the complexity of assessing and managing trauma cases in older adults, including the impact of physiological changes on injury outcomes, challenges faced by ambulance workers, and the lack of a robust trauma care infrastructure in Saudi Arabia. Moreover, it identifies gaps in geriatric-specific training and prehospital care pathways that limit effective management. To address these issues, recommendations include enhancing geriatric-specific education for ambulance workers, improving trauma system infrastructure, and conducting further research to explore the impacts of aging on trauma outcomes. These efforts are critical for reducing the healthcare burden and improving trauma care quality for older adults in Saudi Arabia.

## 1 Introduction

In recent years, the growing aging population has led to a significant rise in the demand for prehospital services globally, including in Saudi Arabia. This shift has resulted in increased resource allocation to meet the specific needs of older adults (1, 2). In Saudi Arabia, as in other nations, the provision of trauma care for older patients is becoming an increasingly critical aspect of emergency medical services. Despite the efforts of ambulance workers to effectively assess and manage trauma cases involving older patients, providing comprehensive and specialized care for these individuals presents numerous challenges (3–5). The unique characteristics of trauma incidents among older adults, such as falls from lower heights and road traffic collisions, complicate emergency response efforts and contribute to high rates of disability and mortality (6, 7).

The Saudi healthcare system faces distinct challenges in providing trauma care for older adults due to factors such as rapid population growth, an insufficiently developed trauma care infrastructure, and limited geriatric-specific prehospital training. This highlights the importance of understanding the current state of trauma care for older people in Saudi Arabia. Developing a comprehensive understanding of the characteristics of older trauma patients, the existing protocols for managing their care, and the outcomes associated with prehospital interventions is critical for improving patient outcomes and reducing the burden on healthcare services (8). The aim of this review is to highlight the current status of trauma care for older adults in Saudi Arabia, focusing on the unique challenges faced by emergency medical personnel. By shedding light on the consequences of aging for healthcare services, outlining the implications for clinicians, and examining the gaps within the trauma system, this review seeks to enhance the understanding and preparedness of emergency clinicians, ultimately paving the way for future improvements in the quality of prehospital trauma care for older adults.

## 2 Methods

This narrative review synthesized current literature to evaluate the status of trauma care for older adults in Saudi Arabia. The methodology is described across the following subsections.

### 2.1 Sources of information

The review utilized PubMed and Google Scholar, selected for their extensive coverage of biomedical and health sciences research and their multidisciplinary approach.

### 2.2 Keywords and search strategy

The literature search was guided by keywords and phrases such as “trauma care,” “older adults,” “prehospital care” and “Saudi Arabia.” Articles were selected based on their relevance to prehospital trauma care and the challenges specific to older adults in Saudi Arabia. Boolean operators (e.g., AND, OR) were employed to combine search terms with synonyms and refine the results.

### 2.3 Time frame and language restrictions

Papers published between 2004 and 2024 were included in the search to ensure the selection of the most relevant and recent studies. Only English-language pieces were included to ensure consistency and clarity in the reviewed literature and align with the authors’ language proficiency.

### 2.4 Types of literature

The review included relevant peer-reviewed journal articles, systematic reviews, and government reports.

### 2.5 Screening process

Initially, papers were screened based on titles and abstracts to exclude irrelevant pieces. Subsequently, full-text reviews were conducted to assess eligibility based on relevance to the review objectives.

### 2.6 Integration and analysis

Relevant pieces were thematically analyzed to identify gaps in the trauma care system, focusing on geriatric-specific challenges, prehospital services, and infrastructure issues. Key themes were developed iteratively, guided by the overarching aim of improving trauma care outcomes for older adults.

### 2.7 Synthesis of findings

Data were synthesized into a narrative format, aligning with the review’s goals to highlight unique challenges, draw comparisons with international systems, and propose evidence-based recommendations for improving Saudi Arabia’s trauma care and infrastructure.

### 2.8 Peer review

The data were reviewed internally by the co-authors and both internally and externally by academics with interests and expertise in emergency care. This peer-review process ensured the accuracy, clarity, and relevance of the findings, which were refined to align with current evidence and best practices.

### 2.9 Data and reference management

We used Mendeley literature management software to systematically document and organize detailed records of our search results, screening processes, and references.

## 3 The global aging population

The global population is experiencing rapid aging due to advancements in chronic disease management, healthier lifestyles, and improvements in living conditions such as housing and nutrition. While these factors have led to longer life expectancy, they have also resulted in a rise in injuries among older adults (4, 9–11). Bala et al. (12) estimated that the global population of older adults will reach 2 billion by 2050, based on statistics from the World Health Organization. Studies on population demographics show that by 2030, 20% of the United States’ population will be over the age of 65. Similarly, 23% of the United Kingdom’s population will be over 65 by 2035, with Europe and Australia projecting that 30 and 21% of their populations will be over 65 by 2050 and 2054, respectively (4, 13–15).

Aging has been identified as a key factor contributing to the increased incidence of injuries among older adults (16, 17). This trend has led to growing demand for ambulance services and emergency department care (18), placing additional challenges on ambulance personnel when attending to injured older adults (19). In the United States, older patients represent 25% of all injury-related hospital admissions (20), reflecting a significant and increasing burden on healthcare systems. As a result, healthcare costs, both in prehospital and in-hospital settings, are expected to rise due to increased complication rates and extended hospital stays (20–22).

## 4 The implications of aging changes for older people, healthcare services, and clinicians

This section outlines three categories of implications resulting from injuries sustained by older patients.

### 4.1 The implications of aging changes for injured older patients

Assessing and managing older patients with injuries presents more complexity compared to younger patients (23). Research indicates that older victims of low-velocity vehicle accidents have significantly higher fatality rates than younger victims (24, 25). Similarly, Hashmi et al. (26) noted that older adults are more susceptible to injury despite experiencing similar injury mechanisms as younger people. For examples, older patients often deteriorate rapidly after injuries due to factors such as reduced cardiac reserve, increased comorbidities, and impaired compensatory mechanisms, ultimately leading to poor outcomes (16, 27).

One of the major areas of concern is traumatic brain injury (TBI) in older adults. Two main factors contribute to the increased risk of TBI among this population. Firstly, the dura becomes more adherent to the skull with age, and secondly, the increased use of aspirin and anticoagulant therapies for chronic conditions heightens the risk of bleeding (28). Additionally, normal age-related changes, such as cerebrovascular atherosclerosis and decreased free radical clearance, increase susceptibility to injury and the rate of oxidative damage after TBI (29). Head computed tomography (CT) in older adults may reveal findings of moderate brain atrophy even when neurological examination appears normal, further complicating assessment and management (29).

### 4.2 The implications for services providing care for injured older patients

The implications for healthcare services can be further divided into two key areas: (a) implications of geriatric care for healthcare services and (b) implications of injury prevention for healthcare services.

#### 4.2.1 The implications of geriatric care for healthcare services

Older patients with injuries are often transported by ambulance services to trauma units or major trauma centers (MTCs) where they receive prolonged care, including social and healthcare services, until rehabilitation or early discharge (30). Studies suggest that comorbidity and mortality rates are significantly higher for injured older patients compared to younger ones, posing additional challenges for healthcare services (10, 30). This increased burden arises from greater resource demands, prolonged hospital stays, and the need for costlier care, all of which reduce healthcare system efficiency (21, 31, 32).

The financial burden associated with geriatric trauma care represents a growing challenge for healthcare systems worldwide. Approximately 30% of trauma expenditures in the US are attributed to geriatric trauma, amounting to an estimated 9 billion US dollars in 2009, with projections indicating continued growth (33, 34). Falls, motor vehicle collisions (MVCs), and pedestrian-related incidents are the leading causes of injury among older adults, often resulting in higher injury severity, undertriage, and case fatality rates than in younger individuals (33). Prevention strategies, if appropriately applied through structured and well-planned clinical pathways, could contribute significantly to reducing costs (33).

#### 4.2.2 The implications of injury prevention for healthcare services

Preventive programs to minimize injuries in older adults have other implications for healthcare services. Identifying both intrinsic and extrinsic risk factors is crucial to guide effective preventive solutions (35). Intrinsic factors include age, multiple illnesses, musculoskeletal disorders, history of falls, cognitive and visual impairments, depression, fear of falling, frailty, balance disorders, and polypharmacy (36). Extrinsic factors include environmental risks such as slippery floors, poor lighting, electrical cords, unsuitable footwear, and slippery handrails (36). A multifactorial approach targeting both intrinsic and extrinsic risk factors is recommended to reduce injuries in older adults (37).

Preventive programs often involve exercise, care planning, medical interventions, modifications to the physical environment, educational initiatives, and medication reviews (38, 39). However, some authors argue that implementing such multifactorial strategies can be costlier than the healthcare costs associated with injuries (40). Developing countries may face additional challenges in effectively managing falls due to high associated costs (41). Thus, effective and cost-effective preventive strategies are needed to mitigate financial implications for healthcare services.

### 4.3 The implications of aging changes for clinicians

As older patients increasingly visit emergency departments (EDs), clinicians face additional challenges related to inadequate training and the complexity of these cases (42–44). A qualitative study has highlighted several barriers to caring for older patients with dementia, such as difficulties in diagnosis, insufficient understanding of dementia, and inadequate geriatric education (45). Although some geriatric-specific care guidelines have been developed, there is limited evidence supporting their effectiveness in practice (46).

Nurses and ambulance workers face similar challenges. Inadequate geriatric education for nurses has been linked to suboptimal care and reduced patient satisfaction (47, 48). For ambulance workers, aging-related challenges in caring for injured older patients can lead to long-term disabilities or even death. Despite the evidence of aging impacts, ambulance workers are still less likely to transport high-risk older patients to definitive care facilities due to a lack of specific geriatric training (19, 49–51). Moreover, insufficient training in dealing with older patients can prevent paramedics from applying holistic care (52). Addressing these gaps through improved education and training for clinicians and ambulance workers is crucial to enhance the quality of trauma care for older patients.

## 5 Recent estimates of injuries/deaths among older cases admitted to Saudi hospitals

Saudi Arabia is the largest country in the Middle East, covering approximately 2,150,000 square kilometers, which is twice the size of the United Kingdom, France, and Germany combined (53). The population in Saudi Arabia has grown rapidly, with an average annual growth rate of 2.38%, reaching a total population of 34,110,821 across the 13 Saudi regions (54). In Saudi Arabia, 72% of the population falls within the 15–64 age group, 24.5% are under 15, and only 3.5% are over 65 (54). Reporting and comparing injury and death rates as proportions of the population, as seen in countries like the United Kingdom and the United States, can provide valuable insights. However, the current estimates from the Saudi Ministry of Health only present raw numbers without providing percentages or rates that would offer a clearer understanding to the readers. Furthermore, these estimates focus solely on motor vehicle collisions (MVCs), despite the existence of other injury mechanisms, as MVCs remain the leading cause of mortality and disability among trauma patients in Saudi Arabia according to World Health Organization statistics (55). The lack of a national trauma database in Saudi Arabia poses a significant challenge in identifying and estimating all trauma cases and their respective mortality rates (55). Alferdaus and Shaher (2021) reported that some Saudi hospitals do not provide comprehensive data on injuries, highlighting the absence of an injury surveillance mechanism or injury registry, as well as the lack of quality assurance measures. They further noted that trauma research in Saudi Arabia is limited, primarily due to the lack of a trauma registry (56).

Most recent trauma-related studies in Saudi Arabia have emphasized the burden of MVCs on both younger and older populations. For example, Abolfotouh et al. (2018) found that 52% of 3,786 injured patients admitted to the King Abdulaziz Medical City (KAMC) trauma center were MVC cases, followed by 25% who were fallers (72). Another study showed that 81.2% of 335 trauma patients admitted to KAMC were involved in MVCs, followed by 9.6% involved in pedestrian collisions, with fallers accounting for 3.9% (57). The emphasis on MVC data by the Ministry of Health may be an attempt to address the economic impact of MVCs, which account for 4.7% of the gross financial product and exceed 5.85 billion US dollars annually, according to government statistics (55, 58).

## 6 The trauma system in Saudi Arabia: current status and gaps

A trauma system is defined as a planned, systematic, and coordinated network that integrates all facilities with the capacity to manage injured patients, involving collaboration with the local public health system (59). It comprises four key components: injury prevention, prehospital care, acute care units, and post-hospital care (60). Injury prevention is the first component, aimed at reducing disability and mortality rates by focusing on contributory factors that increase mortality and identifying high-risk age groups (61). The Saudi government has made significant efforts to implement and improve public safety and high-quality healthcare to mitigate injury events (62). Despite these efforts, the rate of mortality and trauma events remains high, imposing substantial burdens on the government and healthcare systems (62).

Two recent Saudi studies have indicated that motor vehicle collisions remain the leading cause of death among trauma patients (63, 64). Effective emergency care in the trauma system involves the coordination of communication, activation of healthcare teams, and implementation of interventions at the scene. These actions include managing resources and providing care until patients are transported to a definitive care facility (59). The concept of the “golden hour” highlights the importance of swift trauma management from the scene to hospital arrival (59).

The acute care and post-hospital care components focus on delivering multidisciplinary interventions to manage major trauma patients from the emergency department (ED) until rehabilitation (62). In Saudi Arabia, large cities typically have one or two tertiary hospitals that manage major trauma patients, with only two trauma centers (King Saud Medical City KSMC and King Abdulaziz Medical City KAMC in Riyadh) maintaining trauma registries and meeting the criteria for level I trauma centers (55, 65). Post-hospital care involves rehabilitation that aims to restore mental and physical function, reduce disability, and improve patient independence (59). There are currently three rehabilitation centers in Saudi Arabia under the Ministry of Health administration (55).

A recent study argued that the trauma system in Saudi Arabia is currently unorganized and lacks integration, making it inadequate for the complexity and urgency of trauma care (66). The vast geographical distances, harsh climate, and lack of communication infrastructure are among the key obstacles hindering the development of a national trauma system. The Saudi trauma system currently faces several challenges: (1) Variability in trauma care across regions and differences in trauma care expertise (67). (2) Only two centers in Riyadh qualify as level I trauma centers, leaving other regions without sufficient major trauma centers (56). (3) Lack of a formal trauma registry for collecting and analyzing data to improve trauma care (56). (4) No clear guidelines for hospital referral, which contributes to ambiguity regarding the role of tertiary hospitals in treating trauma patients (55). These issues contribute to the overall difficulty of understanding patient characteristics and outcomes following injury events due to the absence of a robust national trauma system (67).

Furthermore, (5) the trauma system in Saudi Arabia lacks efficient prehospital networking, including connecting prehospital triage information with receiving hospitals and integrating data across prehospital and in-hospital settings (67). (6) The absence of clear trauma activation protocols aligned with the American College of Surgeons (ACS) criteria and (7) the lack of research investments further exacerbate challenges in developing standardized care protocols (55). (8) Trauma bypass and field triage guidelines are missing in Saudi Arabia, which hinders optimal decision-making regarding the level of care and destination of trauma patients (55).

To address these gaps, Saudi Arabia has established a clinical advisory group for trauma care as part of its Vision 2030, involving multidisciplinary members from EMS, emergency and disaster management, emergency departments (ED), trauma surgery, nursing, pediatric emergency, trauma research, and rehabilitation (66). However, challenges such as a shortage of transport resources, lack of collaborative networks, and inadequate rural trauma care coverage remain to be addressed before the full-scale implementation of an integrated trauma care system can be realized (66).

## 7 The current status of prehospital care in Saudi Arabia

Prehospital care in Saudi Arabia is provided primarily by the Saudi Red Crescent Authority (SRCA), established in 1934 and funded by the government, which offers free healthcare both domestically and for humanitarian relief efforts abroad (55). The emergency medical services (EMS) system follows an Anglo-American model, with ambulances staffed by trained Emergency Medical Technicians (EMTs) and Paramedics employed by the SRCA (68). The formalization of EMS education began in the early 2000s, focusing on improving service quality and reinforcing public trust. The Emergency Medicine Institute in Riyadh, established in 2002 by the Saudi Council for Health Specialties, remains the first and only specialized institute for training EMS personnel (68). Subsequently, Saudi universities have introduced Bachelor of Science programs in EMS, reflecting the rising demand for skilled and qualified personnel (68).

Despite these developments, recent studies have indicated significant gaps in EMS personnel knowledge, including awareness of critical symptoms such as those related to stroke (69). This gap highlights the need for improved educational programs and screening tools (69). AlShammari et al. (70) argued that this lack of knowledge is indicative of underdeveloped EMS education in Saudi Arabia, pointing to a need for broader curriculum development and enhanced training.

Training remains the most crucial component of prehospital care, as it equips EMS personnel to handle emergencies effectively (59). EMTs provide basic life support, including skills like airway management, cardiopulmonary resuscitation, automated defibrillator use, hemorrhage control, and rapid patient transport (55). Paramedics, on the other hand, are trained to deliver advanced life support, which includes administering medications, handling high-risk trauma, and managing major incidents (55). Due to their higher skill levels, the SRCA prefers hiring paramedics with bachelor’s degrees, typically trained in Saudi universities.

Continuous education is mandatory for EMS personnel, who undergo courses such as Basic Life Support (BLS), Prehospital Trauma Life Support (PHTLS) or International Trauma Life Support (ITLS), and Advanced Cardiac Life Support (ACLS), as required for professional accreditation by the Saudi Commission for Health Specialties (55). However, despite the availability of these courses, EMS personnel in Saudi Arabia are still not meeting the local and national needs in terms of competency and skills (59).

The evolution of EMS education in Saudi Arabia can be categorized into three stages: (1) workplace training of first aid providers (1934–2005), (2) the professional technician diploma (2005–2012), and (3) the academic transition to a bachelor’s degree in EMS (2007–present) (70). As for trauma care education, its goal is to enhance knowledge, skills, attitudes, and professional relationships (71). However, it is still unclear whether existing trauma training programs significantly affect trauma-related mortality outcomes (62). Research is needed to evaluate how trauma training in Saudi Arabia impacts healthcare providers’ competencies, confidence, and trauma outcomes, as no data currently exists to confirm these effects (62).

## 8 Saudi vision 2030 and the future of trauma care

The national clinical advisory group for trauma care which operates under the Vision Realization Office of the Ministry of Health, aiming to develop a comprehensive trauma care system in Saudi Arabia (66). Several workshops have been conducted with over 300 healthcare professionals in Riyadh to assess the new trauma system, which is set to be initially implemented in the Riyadh region (66).

These workshops identified several gaps that hinder the development of a trauma care system in Saudi Arabia, including insufficient prehospital transport resources and capabilities, the absence of collaborative networks, hospital transportation limitations, inadequate staffing, a public awareness gap for rehabilitation and community discharge, limited trauma care networks in rural areas, and shortages in pediatric trauma care centers (66). As a result, an initial standard trauma care pathway was proposed, which will first be implemented and refined in Riyadh before regional application (66).

It is noteworthy that most trauma-related studies in Saudi Arabia have primarily focused on the general trauma population. A recent study highlighted that high rates of mortality and trauma incidents continue to impose significant burdens on the government due to increased healthcare costs (62). Moreover, another Saudi study reported increased in-hospital mortality and disability among trauma patients and recommended preventive injury measures to mitigate these issues (72).

## 9 Conclusion

The trauma care system in Saudi Arabia faces significant challenges due to the complexities involved in managing the aging population and the unique needs of older patients. Despite the growing elderly population, the healthcare infrastructure remains insufficient, with gaps in the trauma care pathways, such as the absence of robust prehospital transport resources, collaborative networks, and specialized trauma centers, which hinder effective management and timely interventions. The Saudi Vision 2030 presents a promising future for an improved and integrated trauma care system. However, before its full-scale implementation, there is an urgent need to address the specific gaps identified during workshops, such as limitations in transportation, rural trauma care capabilities, and community rehabilitation services. These measures are essential to ensure that the trauma care system can respond effectively to the increasing demand for geriatric-specific services. It is crucial that ambulance workers receive enhanced geriatric-specific education to expand their roles and make high-level decisions for older patients. Furthermore, comparing the Saudi system with international examples, such as the United Kingdom’s trauma system, shows the necessity of establishing a comprehensive trauma registry, prehospital protocols, and trauma activation procedures in line with the American College of Surgeons (ACS) criteria. Such systems have been shown to significantly improve patient outcomes and reduce mortality rates in similar contexts.

The increasing burden of trauma among older people necessitates strategic policy interventions in Saudi Arabia. Establishing a national trauma registry would be a significant step in collecting and analyzing data to improve trauma care quality. The introduction of clear referral guidelines between hospitals, prehospital networking for triage, and training modules based on the ACS guidelines would also contribute to more coordinated and effective care. Moreover, research focusing on understanding the impact of aging changes on prehospital care and identifying factors that contribute to a lack of awareness among ambulance clinicians will be crucial to closing existing knowledge gaps. In summary, trauma in older people increases the healthcare burden in Saudi Arabia, and the age factor has a substantial impact on trauma care. Research is required to identify the impacts of aging changes on prehospital care and patient outcomes in Saudi Arabia and explore factors that lead to a lack of awareness of ambulance clinicians regarding these impacts. By adopting a structured approach, incorporating international best practices, and investing in targeted education and system improvements, Saudi Arabia can significantly enhance trauma care outcomes for its growing elderly population, ultimately improving the quality of care and reducing the healthcare burden on the system.
